# Advances in the assessment of cosmetic outcomes, sensory alteration in surgical areas, and health-related quality of life of endoscopic thyroidectomy

**DOI:** 10.1186/s12957-024-03307-7

**Published:** 2024-02-13

**Authors:** Di Zhou, Zeyu Zhang, Xiaolin Dou, Fada Xia, Xinying Li

**Affiliations:** Division of Thyroid Surgery, Department of General Surgery, Xiangya Hospital of Central South University, Changsha, China

**Keywords:** Endoscopic thyroidectomy, Thyroid diseases, Cosmetic outcome, Sensory alteration, Quality of life, Assessment

## Abstract

**Background:**

Endoscopic thyroidectomy has been preliminarily proven effective and safe for thyroid diseases. The cosmetic outcomes and life quality are critical contents of postoperative assessment. This review will primarily focus on the assessment methods and results related to cosmetic outcomes, sensory alteration of surgical area, and quality of life following endoscopic thyroidectomy.

**Methods:**

A comprehensive search of published articles within the last decade was conducted using the terms “endoscopic/robotic thyroidectomy,” “patient satisfaction scores,” “questionnaire,” “quality of life,” and “cosmetic” in PubMed.

**Results:**

Assessment methods for postoperative cosmetic satisfaction and sensory alterations encompassed verbal/visual analog scales, scar evaluations, Semmes–Weinstein monofilament tests, and more. The evaluation of postoperative quality of life in endoscopic thyroidectomy involved tools such as SF-36, SF-12, thyroid-specific questionnaires, thyroid cancer-specific quality of life questionnaires (THYCA-QOL), as well as assessments related to voice and swallow function. The cosmetic results of endoscopic thyroidectomy generally surpassed those of open thyroidectomy, while the quality of life in endoscopic procedures was either superior or equivalent to that in open thyroidectomy, especially with respect to general health, role emotion, and vitality.

**Conclusions:**

Assessments of cosmetic outcomes and sensory alterations following endoscopic thyroidectomy predominantly relied on patients’ subjective feelings. The objective and subjective perspectives of scar assessments remain underutilized. In addition, postoperative laryngoscopy and voice function assessments in endoscopic thyroidectomy procedures require more attention.

## Introduction

The anterior cervical approach, also known as the Kocher incision, has traditionally been the gold standard for thyroidectomy [1]. The incidence of thyroid disorders is on the rise, especially among young individuals, predominantly females. Simultaneously, the demand for cosmetic considerations in surgical outcomes, coupled with advancements in surgical tools, has driven the adoption of endoscopic thyroidectomy. Miccoli et al. pioneered the field with a minimally invasive video-assisted thyroidectomy performed through a 2-cm cervical incision [2]. Over the past few decades, various alternative approaches, categorized as “remote access,” have emerged to minimize visible cervical scarring. These approaches include the trans-axillary approach, transoral/vestibular approach, retro-auricular approach, transareola approach, bilateral axillo-breast approach (BABA), subclavian approach, and others [3–8].

The choice between endoscopic and conventional thyroidectomy primarily hinges on several factors, including the patient’s desire for cosmetic results, quality of life considerations, and the recommendations of their surgeons. Additionally, patients often weigh other factors such as surgical risks, long-term complications, overall effectiveness, postoperative discomfort, and the associated expenses [9–13]. Numerous studies have provided evidence supporting the equivalency of endoscopic thyroidectomy conducted through various approaches, to open thyroidectomy in terms of feasibility, safety, and validity [5, 14–17]. Notably, endoscopic thyroidectomy demonstrates a clear advantage in terms of cosmetic outcomes when compared to conventional open thyroidectomy, although the methods of assessment may vary across different research articles. The visual analog scale is frequently employed for evaluating cosmetic outcomes, relying on patients’ subjective perceptions [18–23]. The Vancouver Scar Scale (VSS) and Scar Cosmesis Assessment and Rating (SCAR) are commonly used as objective methods to assess scarring [24–26]. The incidence of sensory alteration is higher in endoscopic thyroidectomy compared to open thyroidectomy. Apart from the postoperative pain in the cervical area, there are new sensory changes in endoscopic thyroidectomy. Numbness in the submental and lip skin, resulting from mental nerve injury, may occur in transoral thyroidectomy via the vestibular approach, whereas paresthesia of the chest area is common in the trans-axillary approach. There have been limited approaches for assessing specific sensory alterations in various types of endoscopic thyroidectomy. Furthermore, the quality of life assessment methods following endoscopic thyroidectomy exhibit a diverse array, with discernible distinctions and intersections. Xuan et al. employed the SF-36 short form to juxtapose the quality of life disparities between transoral thyroidectomy and open thyroidectomy [27]. Li et al., on the other hand, utilized a THYCA-QOL to appraise the quality of life distinction between transaxillary and open thyroidectomy [3]. It is worth noting that certain critical complications closely associated with quality of life, such as voice and swallowing dysfunction, may have been inadvertently overlooked in prior research. Hence, it becomes imperative to establish an appropriate and consistent methodology for assessing outcomes related to cosmetics, sensory changes, and overall quality of life. This review will elucidate the commonly employed assessment methods and present the findings regarding cosmetic outcomes, sensory changes in surgical areas, and quality of life in the context of endoscopic thyroidectomy.

## Materials and methods

**Fig. 1:**
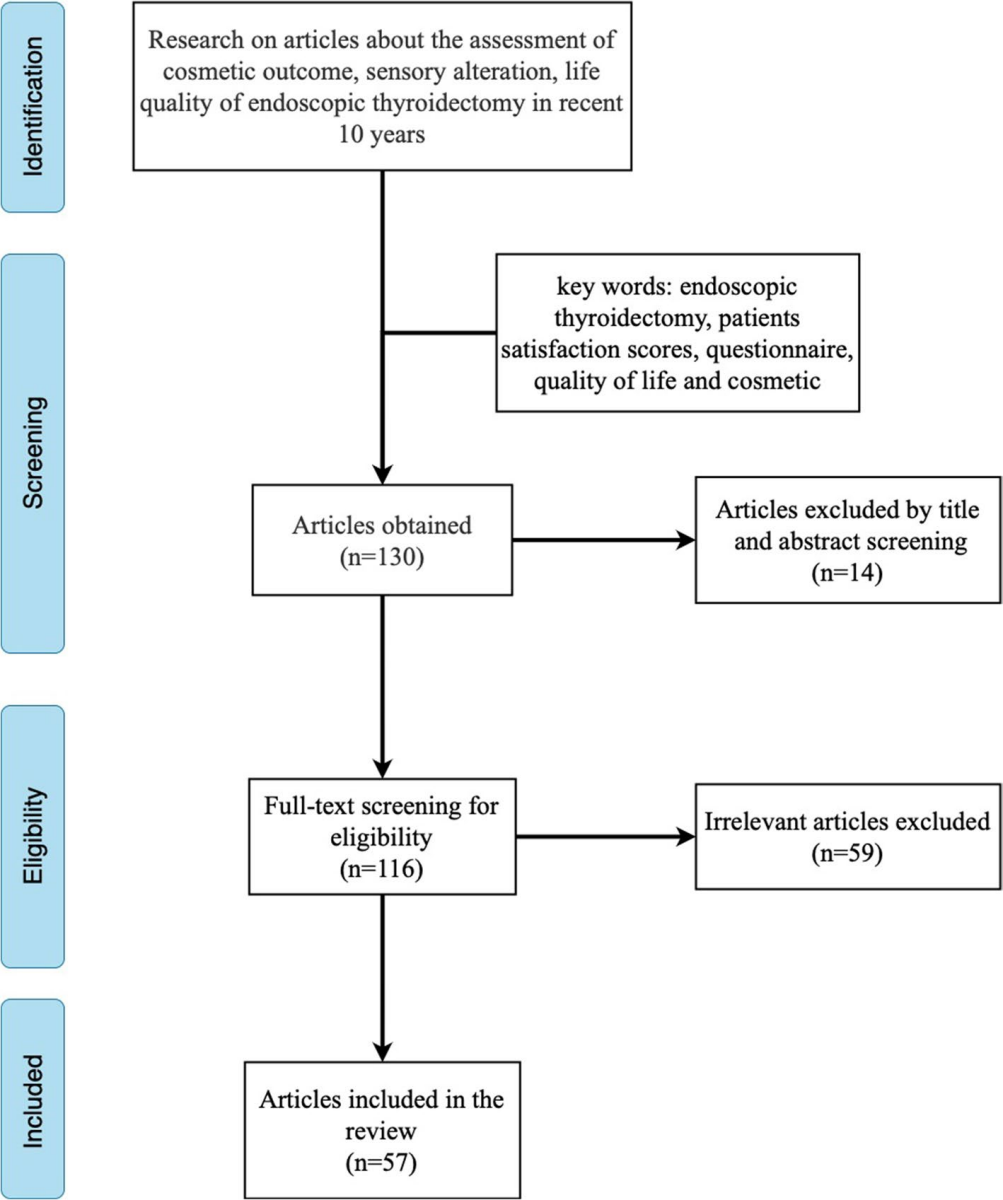
Flowchart of the literature search and study selection process

A comprehensive search of published articles in the past decade was conducted using the terms “endoscopic thyroidectomy,” “patient satisfaction scores,” “questionnaire,” “quality of life,” “robotic,” and “cosmetic” in PubMed. A total of 130 articles were obtained. With 14 reviews and 59 irrelevant articles omitted, 56 directly relevant articles were included in this review (Fig. 1).

## Assessment of cosmetic outcomes

### Method of assessment

#### Visual analog scale/verbal rating scale (VAS/VRS)

Cosmetic outcomes are typically assessed subjectively using a VAS or VRS on a scale ranging from 1 to 10, where a rating of “1” indicates a high level of satisfaction, while “10” corresponds to extreme dissatisfaction [26]. The choice of scale may vary among different studies, with some utilizing scales like 0 to 5 points or 1 to 6 points [27, 28]. Following a designated follow-up period, the mean scores from these scales are compared between different groups to gauge the cosmetic outcomes. Similarly, the severity of scarring can be evaluated using a similar approach. For instance, Yan et al. employed the Vancouver Scar Scale to describe scar severity, with a higher score indicating more severe scarring [26]. Nguyen used a scale of 0 to 5 points to assess paresthesia of the cervical and mandibular area after transoral thyroidectomy [27]. These evaluation systems were generally scored based on the patient’s subjective experience.

#### Scar assessment

The scarring process plays a pivotal role in determining the cosmetic outcomes. Li et al. conducted a study in which they utilized the Patient and Observer Scar Assessment Scale (POSAS) to evaluate scarring in patients undergoing transaxillary thyroidectomy. The patient scale comprises six criteria, specifically evaluating scar color, flexibility, thickness, relief, itching, and pain. The observer scale, on the other hand, focuses on five aspects: scar vascularization, pigmentation, flexibility, thickness, and relief. Each of these criteria was scored on a scale of 1 to 10 [3]. Chen et al. employed a different scoring system known as Scar Cosmesis Assessment and Rating (SCAR) to appraise postoperative scars. This system encompasses six items evaluated by the surgeon, including scar spread, erythema, dyspigmentation, track/suture marks, hypertrophy/atrophy, and overall impression. Additionally, two items are assessed from the patient’s perspective, specifically the presence of itch or pain at the wound site. The total score ranged from 0 to 15 points, and the scale was independently and randomly finished by four surgeons [24]. Ji et al. used the VSS in the study, which consists of four variables: vascularity, pigmentation, pliability, and height. Scores range from 0 to 14, with “0” corresponding to normal skin [25]. Liu et al. utilized the Patients’ Scar Assessment Questionnaire, with scores ranging from 0 to 3 points. A score of “0” indicated no self-concern regarding scarring, while scores of “1,” “2,” and “3” corresponded to slight concern, general concern, and complete concern, respectively [29]. Chen et al. incorporated patient-assessment questionnaires in their research, covering four dimensions: appearance, self-awareness, appearance satisfaction, and symptom satisfaction [30].

### Assessment results

#### Cosmetic results of VAS/VRS

From the cosmetic scoring of the common endoscopic approaches (transoral, transaxillary, retro-auricular, areola/chest and breast, robotic approach). Patients` satisfaction with the postoperative cervical appearance and cosmetic outcomes was significantly higher than that in traditional open thyroidectomy [25, 31–36]. However, when comparing different endoscopic approaches, the distinction in cosmetic performance was not readily apparent. Wirth et al. conducted a study comparing the BABA to the retro-auricular approach, and their findings indicated no significant difference [28]. Similarly, another study evaluated the cosmetic outcomes of the transoral approach versus the chest and breast approach and found no statistically significant distinction between them [19, 32]. Likewise, a comparison between minimally invasive video-assisted thyroidectomy and the BABA approach showed no discernible difference after a 2-year follow-up period [30]. Furthermore, no significant distinction was observed between the trans-axillary and retro-auricular approaches in a separate study [21, 37]. Another investigation that examined the cosmetic outcomes of the unilateral axillo-breast approach and the axillary approach found no noteworthy disparities between the two groups [25].

Collectively, these studies provide compelling evidence that endoscopic thyroidectomy offers superior cosmetic outcomes compared to open thyroidectomy. However, it is noteworthy that no significant differences were observed among various endoscopic approaches. For a comprehensive overview of the assessment methods and results pertaining to cosmetic outcomes, please refer to Table 1. The VAS/VRS are extensively employed in various research studies due to their convenience and simplicity. These scales offer a viable option for researchers to conduct statistical analyses by quantifying patient satisfaction. Nevertheless, these methods come with inherent drawbacks. The subjective nature of the scales poses a challenge, leading to inaccurate results due to variations in individual standards.

**Table 1:** Results of cosmetic outcome and pain assessment

Researchers	Surgery approach	Score scale (points)	No. of cases	Study design	Follow-up time	Result
Yang et al. [38]	(Transoral) Observation group/control group	Pain: 0–10	30/30	Retrospective study^a^	Pre-op, post-op 1 day, 3 days, 7 days	Less pain in intervention group on post-op 3 and 7 days
Yan et al. [26]	Transoral/OT	Cosmetic and pain: 0–10	48/42	Retrospective study	Post-op 3 months	Higher cosmetic satisfaction and less pain in transoral group
Nguyen et al. [27]	Transoral/OT	Cosmetic: 0–5 Pain: 0–10	61/60	Prospective study	Pre-op and post-op 4 weeks, 8 weeks, 12 weeks	Higher cosmetic satisfaction in transoral group and no difference in pain
Wirth et al. [28]	EndoCATS/ABBA/OT	Cosmetic: 1–6	59/52/225	Retrospective study	NA	No difference in cosmetic outcome
Sun et al. [5]	Trans-axillary/OT	Cosmetic: 1–4 Pain: 0–3	105/105	Retrospective study	Cosmetic: post-op 1 month Pain: post-op 1 week, 1 month	Higher cosmetic satisfaction and more pain in transoral group
Piromchai et al. [4]	Submental/OT	Cosmetic and pain: 1–10	24/24	Retrospective study	NA	No difference in cosmetic outcome and pain
Lian et al. [39]	(Anterior cervical approach) MT/MR	Cosmetic and pain: 1–10	46/44	Retrospective study	Post-op 12, 24 h/ NA	More pain for MR group postoperative 12 h and no difference in 24 h; no difference in cosmetic outcome
Bo et al. [31]	Transthoracic/OT/thermal ablation	Cosmetic and pain: 1–10	129/320/56	Retrospective study	Post-op 1, 3, 6, 12 months	Better cosmetic outcome in thermal ablation than OT group
Wongwattana et al. [19]	Transoral/axillo-breast	Cosmetic and pain: 1–10	11/11	Retrospective study	Cosmetic: post-op 90 days Pain: post-op 1, 2, 3 days;	More pain in transoral group; no difference in cosmetic outcome
Nguyen et al. [20]	Transoral/unilateral axillo-breast	Pain: 1–10 Cosmetic: 3 levels^b^	51/50	Retrospective study	Pain: post-op 1, 4, 7 days/NA	More pain in transoral group; no difference in cosmetic outcome
Liu et al. [29]	Transoral/OT	Cosmetic: 0–3	96/425	Retrospective study	Post-op 3 months	Better cosmetic outcome in transoral group
Yuliang et al. [40]	Axillary-breast-shoulder	Cosmetic: 1–4	42	Cohort study	Post-op 6 months	Basically satisfied or very satisfied
Lee et al. [37]	Trans-axillary/postauricular/OT	Cosmetic: 1–5	50/50/50	Retrospective study	Post-op 3 months, 1 year	Better cosmetic outcome in trans-axillary and postauricular group
Johri et al. [41]	BABA/OT	Cosmetic: 1–5 Pain: 1–10	43/60	Prospective study	Pre-op, post-op 6 months	More pain and better cosmetic outcome in BABA group
Sung et al. [21]	GUA/postauricular facelift approach	Cosmetic: 1–5 Pain: 0–10	45/20	Retrospective study	Cosmetic: post-op 1 week, 3 months Pain: pre-op, post-op 1, 3 days, 1 week	No difference between two groups
Song et al. [22]	Robotic/conventional surgery	Cosmetic: 1–5 Pain: 0–4	25/66	Retrospective study	Post-op 1 day, 1 week, 1 month, 3 months	Better cosmetic satisfaction in robotic group More pain in robotic group post-op 1 day and 1 month
Zhu et al. [23]	Transareola	Cosmetic: 0–10 Pain: 0–5	12	Retrospective study	Cosmetic: 2 months Pain: post-op 24 h	High cosmetic satisfaction and median pain
Ryu et al. [35]	Trans-axillary/OT	Pain: 0–10	45/45	Prospective study	Post-op 4 h, 1, 2, 3, 10 days	Lower pain scores in robotic group at post-op 1, 2, and 3 days

Abbreviation: *OT* open thyroidectomy, *MT* muscle transection, *MR* muscle retraction, *NA* not available, *BABA* bilateral axillo-breast approach, *EndoCATS* Endoscopic Cephalic Access Thyroid Surgery, *ABBA* axillo-bilateral-breast approach, *GUA* gasless unilateral axillary

^a^Observation group received neck and face management

^b^Cosmetic outcome was divided into three levels: satisfied, average, dissatisfied

#### Scar assessment results

In addition to assessing patient’s satisfaction, the evaluation of surgical scars provides an objective measure of cosmetic outcomes. Several articles have addressed scar assessment in the context of various endoscopic thyroidectomy procedures. This assessment process involves three key perspectives (patients, observers, and surgeons) which enhance the reliability and validity of the evaluation. For instance, Li et al. conducted a comparative study between the trans-axillary approach and open thyroidectomy, employing the POSAS. According to the research, the patients noted reduced pigmentation in axillary scars and increased suppleness. From the observer’s perspective, the scars exhibited subtler characteristics and decreased hypertrophy. Consequently, the trans-axillary approach demonstrated superior cosmetic outcomes compared to the open approach [3]. Chen et al. conducted an evaluation of postoperative scars using the SCAR to compare the transoral approach with open thyroidectomy. The combined score, which included six items for surgeons and two items for patients, demonstrated a significant decrease in the transoral endoscopic thyroidectomy vestibular approach (TOETVA) group. This suggests that, in the early postoperative period, scar cosmesis is objectively improved with TOETVA in comparison to open thyroidectomy [24]. The evaluation of scars indirectly gauges the cosmetic outcome. By minimizing the impact of subjective variations, the data collected from multiple dimensions become more reliable. Nevertheless, the aspect of patients’ satisfaction with the scar or cosmetic outcome was neglected. Additionally, the use of alternative scales proved to be less convenient compared to the simplicity of VAS/VRS.

## Assessment of sensory alteration

### Method of assessment

#### VAS/VRS for postoperative pain

The VAS/VRS is frequently employed to evaluate postoperative pain following endoscopic thyroidectomy. Comparable to the evaluation of cosmetic outcomes, patients are prompted to designate a numerical value between 1 and 10 to articulate the intensity of pain they are currently undergoing. A rating of “1” signifies no pain or a normal condition, while a rating of “10” indicates severe and intolerable pain. Pain is typically localized in the cervical area and various surgical tunnels in different endoscopic approaches. In a study by Yang et al., the VAS scoring was used to assess pain in patients with cervicalofacial edema and paresthesia following transoral endoscopic thyroidectomy [38]. Several studies have compared postoperative pain levels between endoscopic and open thyroidectomy procedures, as well as different endoscopic approaches, using this pain scale [4, 5, 20, 21, 26, 31, 41, 42].

#### Semmes–Weinstein monofilament test

Yang et al. and Liang et al. conducted assessments of paresthesia in the chin, face, and neck regions of patients who had undergone transoral thyroidectomy using the Semmes–Weinstein monofilament test. The patients were instructed to sit with their eyes closed, after which a nylon filament was applied perpendicularly to the designated area, bent into a C-shape [38, 39]. The size of nylon and its representative in the study were as follows: 2.83 = 0.07 g/mm^2^ (normal sensation), 3.61 = 0.4 g/mm^2^ (hypotactile loss), 4.31 = 2 g/mm^2^ (protective hypoesthesia), 4.56 = 4 g/mm^2^ (protective loss of sensation), and 5.07 = 10 g/mm^2^ (protective loss of sense). A loss of sensation was defined as the inability of patients to perceive the pressure when 10 g was applied [38, 39, 43].

#### Other methods

Liang and colleagues conducted a comprehensive assessment of sensory deficits in the mandibular region following transoral thyroidectomy. Their evaluation encompassed a multi-tiered approach. Initially, a questionnaire was administered, comprising four inquiries pertaining to sensory alterations, the extent of sensory disturbance, recovery timelines, and instances of liquid seepage during ingestion or drinking. Subsequently, the Semmes–Weinstein monofilament test was employed as the second assessment tool. Lastly, the third step involved the administration of the two-point discrimination test to gauge skin sensitivity. This test utilized a two-point stimulus instrument with varying distances, allowing for the quantification of the patient’s ability to discriminate between two separate points. The recorded data captured the minimal distance at which patients could distinguish between these two points [39].

### Assessment results of sensory alteration

Yan et al. observed that postoperative pain following the transoral approach was relatively milder compared to open thyroidectomy [26]. However, Ngeyen et al. reported no discernible difference in pain between transoral thyroidectomy and open thyroidectomy. Several studies have indicated that postoperative pain tends to be more pronounced in transoral thyroidectomy as opposed to open thyroidectomy [20, 24, 44]. Additionally, many studies compared trans-axillary, robotic, chest and breast, and trans-auricular approaches and consistently found that postoperative pain was greater than open in majority [22–24, 45, 46]. Significantly, Sung et al.’s study did not find a substantial difference in postoperative pain between the trans-auricular and robotic approaches [21]. In contrast, Wongwattana et al. compared the transoral approach to the axillo-areola approach and determined that transoral thyroidectomy induced more pain [19]. In summary, endoscopic thyroidectomy typically results in equivalent or increased postoperative pain when compared to open thyroidectomy. Moreover, there are notable variations in postoperative pain across different endoscopic thyroidectomy techniques. The detailed results are summarized in Table 1.

The pain scores were also utilized for comparing the postoperative effects of specific technique enhancements. In their study, Lian et al. compared the outcomes of cutting versus retracting the anterior cervical musculature during endoscopic thyroidectomy, and they observed that the group utilizing muscle retraction experienced more severe short-term pain after surgery [42]. The thoracoscopic approach may lead to damage to the supraclavicular nerve (SCN). In a separate investigation, Zhou et al. investigated the impact of preserving the supraclavicular nerve on postoperative pain in the anterior chest approach. Their findings indicated that the SCN-preserved group exhibited significantly lower levels of sensory disturbance and pain compared to the SCN-damaged group [6]. Yang et al. also discovered that implementing intervention management for cervicofacial edema and paresthesia, which included methods such as ice compress, could expedite the postoperative pain recovery process [6] (Table 1).

The evaluation of sensory alteration also pertains to the surgical incision and the surgical site. Damage to the mental nerve or its branches can lead to mandibular paresthesia. It has been observed that varying degrees of numbness and sensory deficits in the mandibular region following a transoral thyroidectomy typically manifest after the postoperative pain and become more prominent within 1 to 3 months post-surgery. These symptoms typically resolve naturally within 6 months, but a failure to recover may indicate permanent paralysis of the mental nerve [38, 39].

## Postoperative health-related quality of life assessment

### Method of assessment

#### SF-36 short form

The SF-36 short form is a versatile health survey comprising 36 questions. This questionnaire is suitable for assessing postoperative health outcomes and lifelong medical care. The questions are classified into eight domains: physical functioning (10 items), general health (5 items), role-physical (4 items), bodily pain (2 items), social functioning (2 items), vitality (4 items), role-emotional (3 items), and mental health (5 items). Each domain is scored on a scale from 0 to 100, with lower scores indicating a lower quality of life. The SF-36 is the most widely employed tool for evaluating health-related quality of life in patients following thyroidectomy [4, 19, 26–28, 30–32, 34, 44, 46–49].

#### Thyroid surgery-specific questionnaire

The thyroid surgery-specific questionnaire encompassed various aspects such as postoperative numbness and tingling, aesthetic satisfaction, voice dysfunction, swallowing dysfunction, cervical and shoulder movement disorders, physical activity reduction, and psychosocial impairment. Xuan et al. utilized this questionnaire to assess and compare the quality of life between patients who underwent TOETVA and those who underwent open thyroidectomy [27]. The questionnaire comprehensively addressed prevalent postoperative symptoms.

#### Thyroid cancer-specific quality of life questionnaire (THYCA-QOL)

THYCA-QOL was brought up by Husson et al. to assess the postoperative life quality of patients with thyroid tumors in 2013 [50]. This questionnaire comprises 30 questions, each scored on a scale from 1 to 4. In a subsequent study, Li et al. compared the quality of life (QoL) between patients who underwent trans-axillary and open thyroidectomy, using the Chinese version of THYCA-QOL. This version includes 24 questions that assess seven symptom domains (neuro-muscular, voice, attention, sympathetic, throat/mouth, psychological, and sensory symptoms) and six individual scales (scar, feeling cold, tingling sensation, weight gain, headache, and reduced sexual interest) [3].

#### SF-12 short-form

The SF-12 short-form questionnaire evaluates 12 items related to various aspects of an individual’s well-being, including physical functioning, role limitations, physical pain, health perception, energy level, social functioning, and psychological well-being, resulting in physical and mental health composite scores [28]. In their study on transoral and open thyroidectomy, Heede et al. incorporated data from the SF-12 table and also incorporated 13 specific elements from the SF-36 table to assess the QoL of the subjects. It is worth noting that this study did not include a cosmetic satisfaction survey as part of its assessment [44].

#### Assessment of voice function

Digital videolaryngostroboscopy (VLS) stands as the most efficacious method for diagnosing postoperative vocal cord paralysis. This diagnostic procedure allows for direct identification of vocal cord movement disorders through the scope. To evaluate subjective voice disorders, the Vocal Handicap Index (VHI) is employed, consisting of 30 items categorized into three dimensions: physical, functional, and emotional. The questionnaire yields a total score of 120, with each item rated on a scale from 0 to 4. To streamline the assessment process without compromising validity, Rosen et al. introduced the VHI-10, consisting of 10 items [51]. The primary function of the VHI is to gauge the mental, physical, and social impact on patients resulting from voice alterations [52–55].

Acoustic Voice Analysis is a quantitative acoustic assessment of voice quality via software. Patients vocalize a certain vowel, which is recorded by a microphone. The primary parameters are F0 (fundamental frequency), Jitter (the relative variability of the pitch in the short-term, %), PPQ (pitch perturbation quotient), Shim (the relative variability of the peak-to-peak amplitude in the short-term, %), and the APQ (amplitude perturbation quotient), NHR (noise-to-harmonic ratio), MPT (maximum phonation time) [11, 53, 54, 56, 57].

#### Assessment of swallowing function

Swallowing Impairment Index 6 (SIS-6) is a questionnaire including six questions about dysphagia, throat obstruction, and abnormal sensation while swallowing fluids or drugs. Each question is scored from 0 to 4, with a higher score indicating a more pronounced degree of swallowing impairment [4, 53–55]. Barium videofluoroscopy is used to measure the movement of the hyoid bone while swallowing a certain amount of barium paste [53, 58].

#### Other assessments

Qu et al. used a Mental Health Test Questionnaire (DCL-90) to assess the effects of fast-track surgery based on nutritional support on patients’ negative emotions and additionally, they evaluated the postoperative quality of life with a General Quality of Life Inventory (GQOLI-74). The DCL-90 questionnaire comprises nine distinct dimensions: somatization, obsessive–compulsive symptoms, interpersonal relationship, sensitivity, depression, hostility, terror, paranoia, and psychoticism, each assigned a score ranging from 1 to 5. The GQOLI-74 assessment consists of four perspectives: physical, social, psychological, and role, with a maximum achievable score of 100 points [18].

The dermatology life quality index survey reveals the impact of dermatologic issues on patients’ quality of life. The survey includes ten questions, with each question assigned a rating on a scale of 0 to 3 points. A higher score indicates a greater adverse impact on QoL [59, 60].

### Assessment outcomes

#### Outcomes of the quality of life assessment

The SF-36 is the most commonly employed standardized scale for assessing quality of life. After a meticulous selection process focusing on articles providing detailed domain-specific scores, we identified 12 relevant articles. The follow-up duration ranged from immediately after surgery to 2 years. In contrast to open thyroidectomy, endoscopic thyroidectomy yielded a higher postoperative quality of life than its counterpart. Yan et al. studied that the transoral approach scored higher in all eight domains than open thyroidectomy at 3 months postoperatively [26]. Nguyen et al. and Alnehlaoui et al. independently confirmed that transoral thyroidectomy resulted in higher SF-36 scores across all eight domains at 4, 8, 12 weeks, and 6 months post-surgery, as compared to open surgery [27, 47]. The study of Kasemsiri et al. indicated that patients who underwent transoral thyroidectomy exhibited better quality of life in six domains than those who underwent open surgery at both the 6-week and 12-week postoperative intervals [48]. Piromchai et al. carried out a comparative analysis between the submental approach and open surgery, and their findings revealed an improvement in quality of life with the submental approach, specifically in the domains of energy/fatigue, emotional well-being, and general health domains [4]. Additionally, studies employing alternative assessment scales consistently supported the notion that endoscopic thyroid surgery yielded superior outcomes compared to open surgery.

Comparisons of the quality of life among various endoscopic procedures have been a topic of frequent investigation. Wongwattana et al. conducted a study comparing the axilla-breast approach to the transoral approach. They assessed patients using the SF-36 questionnaire both before and after surgery while they were hospitalized. The findings revealed no significant differences in any of the eight domains between these two groups [19]. Shen et al. focused on postoperative QoL of transareola and transoral approach at 2, 4, and 8 weeks postoperatively. Only two domains showed higher scores in the transareola group than in the transoral group [32]. In another study, Chen et al. evaluated the outcomes of the minimally invasive video-assisted thyroidectomy (MIVAT) and BABA procedures, demonstrating that four aspects of the MIVAT group were superior to the BABA group 2 years after surgery [30]. Furthermore, Materazzi et al. investigated the MIVAT approach in comparison to robot-assisted transaxillary thyroidectomy (RATT), revealing that the MIVAT procedure excelled in two aspects but lagged in one [34]. Consequently, it appears that the disparities in quality of life among endoscopic techniques are minimal, and the choice of a specific approach may confer certain advantages (Table 2). The SF-36 and SF-12 short forms are widely utilized for assessing the quality of life, encompassing questions that delve into physical, mental, and social function changes. Nonetheless, a notable limitation of these tools lies in their omission of considerations specific to thyroidectomy-related discomfort and symptoms. In contrast, tailored questionnaires have been developed for individuals with thyroid cancer, such as the Thyroid Surgery-Specific Questionnaire, the University of Washington QOL (UW-QOL) questionnaire, and the THYCA-QOL. These specialized instruments place a particular focus on evaluating symptoms associated with surgery as opposed to providing a comprehensive assessment of overall health.

**Table 2:** The application and results of SF-36

Researcher	Surgery approach	No. of cases	Study design	Follow-up time	Result^a^
Yan et al. [26]	Transoral/OT	48/42	Retrospective study	Post-op 3 months	Higher score of 8 domains in transoral group
Nguyen et al. [20]	Transoral/OT	60/61	Prospective study	Post-op 4, 8, 12 weeks	Higher score of 8 domains in transoral group
Piromchai et al. [4]	Submental/OT	24/24	Retrospective study	Post-op 2, 6, 12, 24 weeks	Higher score of 3 domains in submental group
Bo et al. [31]	Transthoracic/OT/thermal ablation	129/320/56	Retrospective study	Post-op 1, 3, 6, 12 months	No difference among three groups
Alnehlaoui et al. [47]	Transoral/OT^b^	31/28	Retrospective study	Post-op 6 months	Significant improvement of QoL in transoral group
Wongwattana et al. [19]	Transoral/axillo-breast	11/11	Retrospective study		No difference between two groups
Shen et al. [32]	Transareola/transoral	74/57	Retrospective study	Post-op 2, 4, 8 months	Higher score of 2 domains in transoral group
Chen et al. [30]	MIVAT/BABA	60/35	Retrospective study	Post-op 2 years	Higher score of 4 domains in MIVAT group
Kasemsiri et al. [48]	Transoral/OT	32/38	Retrospective study	Post-op 6, 12 weeks	Higher score of 6 domains in transoral group
Bakkar et al. [49]	Transoral	5	Case cohort	Post-op 1 months	High score of QoL in transoral group
Huang et al. [46]	Trans-axillary/OT	75/123	Retrospective study	Post-op 1, 6 months	Better in 3 domains in transoral group
Materazzi et al. [34]	MIVAT/RATT	30/32	Retrospective study		Higher score of 2 domains for MIVAT and of 1 domain in RATT group

Abbreviation: *MIVAT* minimally invasive video-assisted thyroidectomy, *OT* open thyroidectomy, *BABA* bilateral axillo-breast approach, *RATT* robot-assisted transaxillary thyroidectomy

^a^Comparison of 8 domains in SF-36

^b^Comparison of using Intraoperative Indocyanine Green Fluorescence Imaging and Neuromonitoring between two groups

#### Outcomes of voice and swallowing function assessment

Adeyemo et al. conducted a comprehensive assessment of postoperative voice dysfunction in 54 cases of non-malignant goiters. Following surgery, seven patients were diagnosed with positive laryngoscopy findings, while ten patients experienced voice changes in the early postoperative period. Notably, the median VHI-10 score was significantly higher 1 week after surgery compared to baseline. However, at the 3-month mark, the VHI-10 scores of five patients had regressed to baseline levels [52]. In another study by Han and colleagues, the VHI-10 scores of patients who underwent transoral and open thyroidectomy were compared. Interestingly, there were no baseline differences between the two groups at the 3- and 6-month postoperative follow-up [54]. The VHI-10 was also employed to assess the outcomes of different anatomical approaches in endoscopic surgery, revealing similar recovery patterns in VHI-10 scores at 3 and 6 months postoperatively. Notably, the patients’ voices exhibited a certain impact but were largely restored by the 6-month mark [55]. Liu et al. reported their findings regarding the VHI-10 assessment, which demonstrated no significant differences between the BABA group and endoscopic thyroidectomy with respect to preoperative and postoperative scores at 1 week, 1 month, and 3 months [57]. Furthermore, VHI was employed to evaluate the efficacy of intraoperative neuromonitoring (IONM) in safeguarding patients’ voices during robotic thyroid surgery. Notably, the presence of IONM did not yield discernible differences in VHI outcomes between the study groups [61, 62].

Kim et al. researched the possibility of voice pitch preservation in transoral thyroidectomy. Utilizing acoustic voice analysis, the study found no significant postoperative change in fundamental frequency (F0), shimmer frequency (SFF), pitch range, or high pitch between the transoral group and the open surgery group. This suggests that transoral surgery does not impose any additional pitch risk to pitch compared to conventional open surgery [63]. In a related study, Han et al. observed no significant changes in objective acoustic and aerodynamic parameters between preoperative and postoperative assessments for TOETVA and OT approaches [54]. Furthermore, Liu et al. compared the accurate voice outcome between robotic and endoscopic surgery and found that F0 and MPT in the endoscopic group were significantly lower than in the robotic group at 1 week postoperatively [57]. A summary of the methods and results of voice function assessment is provided in Table 3.

**Table 3:** Methods and results of voice function assessment in endoscopic thyroid surgery

Researcher	Surgery approach	No. of cases	Study design	Follow-up time	Assessment method	Result
Liu et al. [57]	Robotic/endoscopic	67/58	Retrospective study	Post-op 1 week, 1 and 3 months	VHI-10, voice acoustic analysis	No significant difference in the VHI-10 scores; F0 and MPT were higher in robotic group post-op 1 week
Kim et al. [63]	Transoral/OT	44/38	Retrospective study	Pre-op, post-op 1 month	TVQ, GRBAS score, laryngoscopy, acoustic analysis	No significant difference in TVQ score, the GRBAS score, or acoustic analysis between two groups
Han et al. [54]	TOETVA/OT	52/50	Retrospective study	Post-op 3, 6 months	VHI-10, GRBAS score, acoustic and aerodynamic analysis	No significant differences in VHI-10, GRBAS scores, acoustic and aerodynamic analysis between two groups
Liu et al. [55]	Subplatysmal/subfascial	129/320/56	Prospective study	Pre-op, post-op 2 weeks, 3 and 6 months	VHI-10	No significant difference in VHI-10 scores between two groups
Lee et al. [61]	Robotic IONM/robotic non-IONM^a^	25/25	Prospective study	Pre-op, post-op 2 weeks, 3 and 6 months	VHI, voice range profile (VRP), laryngoscopy	No significant difference in VHI-10 scores between two groups; earlier recovery in VRP minimum intensity in non-IONM group
Bae et al. [62]	Robotic thyroid surgery^a^	30	Prospective study	Pre-op, post-op 1 and 3 months	VHI-10	The mean VHI-10 score was higher postoperatively and the scores were lower at post-op 3 months than 1 month

Abbreviation: *VHI-10*, voice handicap index 10; *TVQ*, Thyroidectomy-related voice questionnaire; *GRBAS*, the grade of dysphonia, roughness, breathiness, asthenia, and strain; *TOETVA*, transoral endoscopic thyroidectomy vestibular approach; *IONM*, intraoperative neuromonitoring

^a^The studies focused on the outcome of the application of intraoperative neuromonitoring in robotic thyroid surgery

Piromchai et al. reported no difference in swallowing function between submental thyroidectomy and open thyroidectomy [4]. Han et al. likewise observed no statistically significant variance in postoperative swallowing and vocal function when comparing open and transoral surgical approaches [54]. Notably, the retro-auricular approach exhibited superior swallowing outcomes compared to open thyroidectomy. It remains unclear whether this improvement is associated with the retro-auricular approach’s avoidance of linea alba dissection, warranting further investigation. Only two articles provided a similar comparative analysis [53, 55]. In addition, Hyun et al. identified a correlation between swallowing disorders and the use of a muscle flap, as demonstrated in a comparison between gasless trans-axillary and open thyroidectomy [58].

## Conclusion

In summary, the evaluation of cosmetic outcomes, pain levels, and swallowing function following endoscopic thyroidectomy primarily relies on patient-reported assessments. Notably, endoscopic thyroidectomy generally yields superior cosmetic results when compared to traditional open thyroidectomy. After endoscopic thyroidectomy, the pain is typically either greater or comparable to what is observed in open surgery. The commonly employed methods for assessing the overall quality of life in these patients include the SF-36 short form, the SF-12 short form, the thyroid surgery-specific questionnaire, and the THYCA-QOL survey. The quality of life following endoscopic surgery tends to exhibit improvements, or at the very least, is on par with open thyroid thyroidectomy. This is particularly evident in domains such as general health, emotional well-being, and vitality. However, when comparing various endoscopic techniques, no statistically significant differences in overall quality of life emerge. Voice outcome evaluations are typically performed through laryngoscopy in conjunction with the VHI-10 questionnaire. The review has certain limitations that should be acknowledged. It did not specifically focus on the analysis of a particular aspect or assessment tool. Future research endeavors are warranted to conduct a more in-depth comparison of various tools.

Incorporating the perspectives of surgeons, observers, and patients would offer significant advantages in assessing cosmetic outcomes. Moreover, beyond the feasibility and safety of a novel surgical approach, it is imperative to evaluate sensory alterations and quality of life, which is important in shaping patients’ choices regarding their surgical approach and the general applicability of the procedure. The potential damage to the recurrent laryngeal nerve can easily go unnoticed. For endoscopic thyroidectomy, the conventional postoperative measures of laryngoscopy and voice function assessment tend to be insufficient.

## Data Availability

The datasets generated and/or analyzed during the current study are available in the PubMed repository, https://pubmed.ncbi.nlm.nih.gov/.

## Abbreviations

THYCA-QOL: Thyroid cancer-specific quality of life questionnaires
BABA: Bilateral axillo-breast approach
VSS: Vancouver Scar Scale
SCAR: Scar Cosmesis Assessment and Rating
VAS: Visual analog scale
VRS: Verbal rating scale
POSAS: Patient and Observer Scar Assessment Scale
TOETVA: Transoral endoscopic thyroidectomy vestibular approach
SCN: Supraclavicular nerve
QOL: Quality of life
VHI: Vocal Handicap Index
SIS-6: Swallowing Impairment Index 6
DCL-90: Mental Health Test Questionnaire
GQOLI-74: General Quality of Life Inventory
MIVAT: Minimally invasive video-assisted thyroidectomy
RATT: Robot-assisted transaxillary thyroidectomy
IONM: Intraoperative neuromonitoring
OT: Open thyroidectomy
EndoCATS: Endoscopic cephalic access thyroid surgery
GUA: Gasless unilateral axillary
TVQ: Thyroidectomy-related voice questionnaire
GRBAS: The grade of dysphonia, roughness, breathiness, asthenia, and strain
